# Fueling Defense: Effects of Resources on the Ecology and Evolution of Tolerance to Parasite Infection

**DOI:** 10.3389/fimmu.2018.02453

**Published:** 2018-10-31

**Authors:** Sarah A. Budischak, Clayton E. Cressler

**Affiliations:** ^1^W. M. Keck Science Department of Claremont McKenna, Pitzer, and Scripps Colleges, Claremont, CA, United States; ^2^Department of Ecology and Evolutionary Biology, Princeton University, Princeton, NJ, United States; ^3^School of Biological Sciences, University of Nebraska, Lincoln, NE, United States

**Keywords:** tolerance, resistance, resources, foraging, parasite infection, defense strategy

## Abstract

Resource availability is a key environmental constraint affecting the ecology and evolution of species. Resources have strong effects on disease resistance, but they can also affect the other main parasite defense strategy, tolerance. A small but growing number of animal studies are beginning to investigate the effects of resources on tolerance phenotypes. Here, we review how resources affect tolerance strategies across animal taxa ranging from fruit flies to frogs to mice. Surprisingly, resources (quality and quantity) can increase or reduce tolerance, dependent upon the particular host-parasite system. To explore this seeming contradiction, we recast predictions of models of sterility tolerance and mortality tolerance in a resource-dependent context. Doing so reveals that resources can have very different epidemiological and evolutionary effects, depending on what aspects of the tolerance phenotype are affected. Thus, it is critical to consider both sterility and mortality in future empirical studies of how behavioral and environmental resource availability affect tolerance to infection.

## Introduction

Parasite-infected hosts have two, non-exclusive options for mitigating the fitness costs of parasite infection. Resistance describes an individual's ability to reduce its parasite load, while tolerance is a measure of an individual's ability to mitigate the fitness costs of parasite infection without reducing parasite load (1–4). Thus, a more tolerant individual attains higher fitness than others with the same parasite burden. Tolerance can be quantified as the slope of the relationship between parasite load and fitness with a less steep slope indicating higher tolerance [(1) but see (5) for a criticism of this approach]. While the ecological and evolutionary drivers of variation in resistance have been elucidated by decades of studies, variation in tolerance is less well-understood (6–9). In plants, where tolerance in response to damage (e.g., herbivory, infection) has long been studied, the important ecological and evolutionary implications of tolerance have been demonstrated and provide useful parallels for understanding animal host-parasite interactions (10).

Notably, plant tolerance to herbivory depends on environmental resource availability (11). The hypothesis that animal tolerance may also be resource-dependent is supported on general evolutionary grounds; resource availability is a principal selective pressure shaping the evolution of species, as evidenced by decades of studies on resource partitioning and character displacement (12, 13). Moreover, the often strong effects of resources on the ecology and evolution of disease resistance are well-established from both theoretical (14, 15) and empirical (16–19) perspectives. A growing number of animal disease studies suggest that host tolerance to parasites might also be affected by resources [Table 1; (8)]. However, as we review, existing studies often come to mixed conclusions as to the effect of resources on tolerance, suggesting that a theoretical framework is needed to guide hypothesis development and to draw general conclusions.

**Table 1:** Studies of the effects of resources on tolerance show varied outcomes (red, resources reduce tolerance; yellow, resources have no effect on tolerance; green, resources increase tolerance; white, resources affect tolerance).

**Host**	**Parasite**	**Study design**	**Effect of resources on tolerance**	**Tolerance metric**	**Source**
BALB/c and CBA lab mice (*Mus musculus*)	*Heligmosomoides polygyrus* (nematode)	Resource quality (low vs. high-protein diet) crossed with infection status	low quality resources reduce tolerance, but only for BALB/c mice	Fitness proxies (weight gain, intestinal permeability)	(7)
BALB/c lab mice (*Mus musculus*)	*Heligmosomoides polygyrus* (nematode)	Resource quality (low vs. high-protein diet) crossed with single and co-infection status	No effect of resource quality on tolerance	Fitness proxy (weight gain)	(20)
BALB/c lab mice (*Mus musculus*)	*Nippostrongylus brasiliensis* (nematode)	Resource quality (low vs. high-protein diet) crossed with single and co-infection status	Low quality resources reduce tolerance	Fitness proxy (weight gain)	(20)
Blue tits (*Parus caeruleus*)	*Ceratophyllus gallinae* (flea)	Resource acquisition behavior–Flea removal and addition to nests	Behavioral resource supplementation facilitated tolerance	Sterility (offspring quantity and quality)	(21)
Great tits (*Parus major*)	*Ceratophyllus gallinae* (flea)	Resource acquisition behavior–Flea removal and addition to nests	Behavioral resource supplementation facilitated tolerance	Sterility (offspring number and condition, but reduced body size)	(22)
Galápagos mockingbird (Mimus parvulus)	*Philornis downsi (invasive nest fly)*	Resource acquisition behavior–Fly removal from nests	Behavioral resource supplementation facilitated tolerance	Sterility (offspring quantity and quality)	(23)
medium ground finches (Geospiza fortis)	*Philornis downsi (invasive nest fly)*	Resource acquisition behavior–Fly removal from nests	Without behavioral resource supplementation, tolerance was lower	Sterility (offspring quantity and quality)	(23)
Domestic canaries (*Serinus canaria*)	*Plasmodium relictum* (avian malaria)	Resource supplementation crossed with infection	Resource supplementation reduces tolerance	Fitness proxy (hematocrit)	(24)
Cuban tree frog (*Osteopilus septentrionalis*)	*Aplectana* sp. (nematode)	Resource quantity (# crickets) crossed with infection status	Low quantity of resources reduces tolerance	Fitness proxy (weight change)	(25)
Monarch butterflies (*Danaus plexippus*)	*Ophryocystis elektroscirrha* (protozoa)	Resource variation (12 milkweed food plant species) crossed with infection status	Tolerance varies by milkweed species and increases with cardenolide conc.	Mortality (longevity)	(26)
Texas field crickets (*Gryllus texensis*)	*Serratia marcescens* (bacteria)	Resource limitation crossed with infection and wounding	No effect of resource limitation on tolerance	Sterility (egg output) and immune mechanism (glutathione)	(27)
Fruit fliy (*Drosophila melanogaster*)	*Providencia rettgeri* (bacteria)	Resource quality (low vs. high-sugar diet) crossed with infection status and genotype	Lower mortality tolerance on high-sugar diet, but no effect on sterility tolerance	Sterility (# adult offspring produced) and mortality (survival)	(28)
Fruit fliy (*Drosophila melanogaster*)	*Salmonella typhimurim* (bacteria)	Resource quantity (dilute media) crossed with infection status	Resource limitation increases tolerance	Mortality (longevity)	(29)
Fruit fliy (*Drosophila melanogaster*)	*Lysteria monocytogenes* (bacteria)	Resource quantity (dilute media) crossed with infection status	No effect of resource limitation on tolerance	Mortality (longevity)	(29)
Fruit fliy (*Drosophila melanogaster*)	*Escherichia coli* (bacteria)	Resource quality (low vs. high-protein diet) crossed with infection status	Resource limitation increases tolerance, but only during early infection	Sterility (# adult offspring produced)	(30)
Fruit fliy (*Drosophila melanogaster*)	*Lactococcus lactis* (bacteria)	Resource quality (low vs. high-protein diet) crossed with infection status	No effect of resource quality on tolerance	Sterility (# adult offspring produced)	(30)
Fruit fliy (*Drosophila melanogaster*)	*Lactococcus lactis* (bacteria)	Resource quality (low vs. high-protein diet) crossed with infection status	No effect of resource quality on tolerance	Sterility (# adult offspring produced)	(31)
*Daphnia magna*	*Pastura ramosea* (bacteria)	Resource quantity (low vs. high) crossed with infection status and genotype	Low quantity of resources reduces tolerance	Mortality (longevity)	(32)

To date, there have not been any theoretical studies that directly address the question of how resources affect host investment in tolerance to infection (9), where this investment reduces the fitness cost of infection at some cost to the host. We distinguish this theory from other work that has examined how resource-dependent effects on mortality or transmission affect ecological dynamics (15, 33). However, existing theory exploring the implications of investment in tolerance for the ecological and evolutionary dynamics of host-parasite systems does provide indirect insights into how resources might affect tolerance investment. Here we review the empirical studies of resources on tolerance, explore key predictions of existing theory, and discuss how combining theoretical and empirical approaches could further understanding of the effects of resources on the ecology and evolution of tolerance.

## Direct effects of environmental resources on tolerance

### Resources can increase tolerance

Intuitively, tolerance should require host investment of potentially limiting resources to compensate for parasite-induced reductions in host fitness, for example by repairing tissue damage. Support for that intuitive prediction comes from both observational and experimental studies showing that organisms with increased resource consumption have higher tolerance, and that organisms with reduced ingestion have compromised tolerance (Table 1). This evidence comes from studies investigating resource limitation (e.g., low resources vs. “normal”), studies on resource supplementation (e.g., “normal” resources vs. high), or studies of two resource levels, but with no reference to which (if either) is normal for that host in the wild. Notably, the shape of the reaction norm between resources and tolerance cannot be determined from only two resource levels. A such, results from resource limitation studies should not be extrapolated to high-resource conditions, or vice versa. Determining the shape of such reaction norms by quantifying tolerance across a range of resource levels ranging from scarce to over-abundant is a key area for future research.

Numerous observational studies indicate that increasing resource consumption can be a behavioral mechanism of tolerance (21–23). For example, Knutie et al. (23) used a parasite removal experiment to determine that parasitized Galapagos mockingbird nestlings beg more for food and receive increased provisioning from their parents in comparison to their non-parasitized counterparts. The additional resources they received allowed parasitized nestlings to compensate for some of the costs of infection; fledging success was not affected by parasite load. Notably, in the same experiments, the medium ground finch did not increase provisioning to infected nestlings, which resulted in a negative relationship between parasite load and fledging success. Thus, the resource supplementation behavior of mockingbirds makes them more tolerant than the medium ground finch (23). Interspecific variation in tolerance to a generalist parasite could alter transmission dynamics and competition between species, as the tolerant species will support a higher parasite population, fueling spillover infections that drive down the population size of the intolerant host, analogous to the P^*^ concept in apparent competition theory (34). Thus, interspecific variation in tolerance has the potential to affect the ecology and evolution of host communities. Similar forms of “parental compensation” by increasing resource provisioning to parasite-infected nestlings has been observed in other focal bird species (21, 22). Interestingly, although initially broadly accepted, the parallel theory for plant-herbivore-resource interactions, termed the “compensatory continuum hypothesis,” a metaanalysis found little support for the theory (11, 35). For host-parasite interactions, further studies and expanding beyond avian systems may prove useful in determining whether, how commonly, and under what conditions resources and foraging behavior can be used to fuel tolerance.

Moreover, severely malnourished hosts often have diminished investment in both resistance and tolerance defenses (16–19). Resource limitation thus has the potential to result in higher parasite loads and higher fitness costs per parasite. Indeed, Cuban tree frogs show both reduced resistance and tolerance to infection with a parasitic nematode when food abundance is limited (25). If hosts are less able to either resist or tolerate infection, the resulting effects on parasite transmission and host population dynamics may be complex. Individual hosts will have higher load due to reduced resistance, but lower survival and/or reproduction due to reduced tolerance. At the population level, these effects could translate to increased transmission due to higher shedding rates or reduced transmission due to parasite-induced mortality, lower population density, and reduced birth rate of new susceptibles (15).

Even when tolerance responds positively to increasing resource quality and quantity, resistance may not respond similarly. For example, when infected with a bacterial pathogen, the crustacean *Daphnia magna* has increased survival (i.e., higher tolerance) when given high food levels compared to low food levels, despite having higher parasite loads (i.e., lower resistance) at high food levels (32). Likewise, a low-protein diet has been shown to increase resistance but reduce the ability of lab mice to tolerate gastrointestinal nematode infection, when tolerance is measured as a function of weight gain (20) and intestinal barrier function (7). However, the effect of resources on tolerance to nematode infection can vary with host genotype (7); there was no effect of diet on tolerance to infection in a strain of lab mice that better maintained their intestinal barrier during infection. Conversely, genotype did not affect the morality tolerance of bacteria-infected *D. magna* (32). Alternatively, resistance may respond positively to resource quality while tolerance does not; food-limited crickets show reduced resistance but equal tolerance to *ad libitum* fed individuals (27). Taken together, these results indicate that understanding the population-level consequences of resource limitation for disease dynamics will likely require considering the complex interactions among genotype, tolerance, and resistance.

### Resources can reduce tolerance

Reduced resource ingestion is a ubiquitous response to infection across the animal kingdom (36). While initially thought to be a maladaptive side-effect of infection, studies increasingly suggest that illness-induced anorexia may carry benefits for the host (37, 38). For example, fruit flies on a limited (dilute) diet are more tolerant of *Salmonella typhimurim* infections, exhibiting increased fecundity relative to parasite load compared to infected individuals on a standard diet (29). Notably, this beneficial effect of resource limitation on tolerance is infection-specific; diet restriction did not affect tolerance to another bacteria, *Listeria monocytogenes* (29). Similarly, a low-protein diet increases sterility tolerance to *Escherichia coli* infection, but not *Lactococcus lactis* infection in fruit flies (30). A low-sugar diet also increases fruit fly tolerance with respect to mortality due to the bacterial pathogen *Providencia rettgeri*. Interestingly, dietary sugar content does not affect fruit fly fecundity relative to parasite load (i.e., sterility tolerance) (28). Tolerance benefits of a low resource diet are not limited to fruit fly-bacteria pathogen interactions; canaries infected with avian malaria (*Plasmodium relictum*) exhibit higher hematocrit relative to parasite load when on a standard rather than supplemented diet (24). Nonetheless, most studies of infection-induced anorexia have primarily focused on it as a parasite avoidance strategy or a side-effect of resistance responses, leaving anorexia-tolerance relationship a topic warranting further empirical and theoretical attention (39).

## Theoretical predictions for effects of resources on tolerance

### Modeling the evolution of tolerance

Given the limited number of empirical studies on the effects of resources on tolerance to infection, theory may help us understand the implications of these studies and guide hypotheses and design of future empirical research. No prior studies have directly modeled the effects of resources on host investment in tolerance, but existing theory regarding the ecological and evolutionary implications of tolerance investment can be adapted to provide useful, although indirect, insights. In the Appendix in Supplementary Material, we extend existing theory to explicitly account for resources. Analysis of this model shows how the shapes of the relationships between tolerance investment, resources, and host life history can drive the evolutionary response of tolerance to resources.

Here, however, we focus on reviewing existing theory. From a theoretical perspective, tolerance is modeled by assuming that some model parameters (such as virulence) are under the control of both the parasite and the host (40). We will use the following simple model to illustrate many of the conclusions of theory (41, 42):

$$\begin{array}{lll} \frac{dS}{dt} & = & a\left(S+fI\right)-qN\left(S+fI\right)-mS-\beta SI+\gamma I\\ \frac{dI}{dt} & = & \beta SI-\left(\alpha +m+\gamma \right)I \end{array}$$

In this model, *a* is the intrinsic birth rate of the host, *f* is the reduction in intrinsic birth rate due to infection, *q* is the host susceptibility to crowding, *m* is the background mortality rate of the host, β is the transmission rate, α is the virulence (infection-induced mortality rate), and γ is the recovery rate. In this simple model, infection may reduce host fitness by reducing host birth rate (*f*) or increasing mortality rate (α). These two parameters, therefore, depend on both host-specific traits (parameters) and parasite-specific traits. That is, *f* and α are both functions, *f*(*h*_*f*_, *p*_*f*_) and α(*h*_α_, *p*_α_), where *h*_*i*_ and *p*_*i*_ are host and parasite traits, respectively. In a host-centric analysis, *p*_*f*_ and *p*_α_ are assumed to be constant. Finally, investment in tolerance by the host (increasing *h*_*f*_ or *h*_α_) must come at some cost to other aspects of host fitness (otherwise, infinite investment will always be favored). Typically, theory assumes that investment in *mortality tolerance* (*h*_α_) reduces intrinsic birth rate (*a* is a decreasing function of *h*_α_, *a*(*h*_α_)), whereas investment in *sterility tolerance* (*h*_*r*_) increases background mortality rate (*m* is an increasing function of *h*_*r*_, *m*(*h*_*r*_)). Importantly, sterility tolerance has no effect on parasite fitness, whereas mortality tolerance increases parasite fitness (43, 44). This distinction has important consequences for both ecological and evolutionary dynamics.

This sets up the basic model for studying the ecological and evolutionary consequences of tolerance. There is also a significant body of research studying “resistance” strategies of host defense (43), such as avoidance (host traits affecting β) or recovery (host traits affecting γ). In these models, there will be trade-offs between host investment in resistance and host intrinsic birth rate.

There are several models that explicitly consider how investment in resistance and tolerance change simultaneously (40, 45) including models that assume a trade-off in investment (44). We will also discuss models that consider the coevolution of hosts and parasites. In these models, parasite traits also vary and are involved in parasite fitness trade-offs (e.g., increasing *p*_α_ increases both infection-induced mortality α and transmission rate β).

Existing theory typically studies the *evolution* of tolerance using evolutionary invasion analysis (46). This framework conceptualizes evolution as a series of mutation events, where “mutant” hosts with new trait values attempt to invade a population of “resident” hosts at their epidemiological equilibrium. If the mutant can invade, it does and the trait composition of the population changes. Ultimately, the theory is seeking to find evolutionarily stable traits; such a trait is a fitness maxima and a host population with that trait cannot be invaded. Other interesting outcomes are possible, such as evolutionary bistability (the existence of multiple evolutionarily stable trait values, only one of which will be attained) and evolutionary branching (evolution of polymorphism in trait values) (46). However, though these predictions tend to be evolutionary, we can also use them to infer how tolerance will change plastically in response to host, parasite, or environmental factors, such as resources. Perfect adaptive plasticity should adjust investment in tolerance in response to changes in the environment such that the population remains at a fitness maximum. Thus, we will assume that predictions for the evolution of tolerance can guide predictions about plastic changes in tolerance as well.

### Implications of mortality vs. sterility tolerance

Before delving into specific predictions of theory, and their potential implications for the effect of resources on tolerance, there is an important distinction to be made between mortality tolerance and sterility tolerance. Studies of mortality tolerance (42, 44, 47–54) vastly outnumber studies of sterility tolerance (40, 44, 55, 56). Mortality tolerance will increase parasite fitness by increasing the host lifespan while infected. As such, investment in tolerance increases parasite fitness, thereby increasing parasite prevalence and hence, the selection for investment in tolerance, driving tolerance to fixation via positive frequency dependence (44, 49). This is in contrast to defense mechanisms that directly reduce parasite fitness: investment in such resistance mechanisms reduces parasite fitness, thereby reducing infection prevalence and, hence, selection for investment in resistance. This negative frequency dependence can lead to other evolutionary outcomes, such as polymorphism in resistance investment (44, 56). Such polymorphism is, in general, impossible in models of mortality tolerance (54). Sterility tolerance, however, can generate such negative frequency dependence because parasite fitness is reduced via the trade-off between sterility tolerance and host background mortality rate. As such, polymorphism is possible, meaning that hosts with both high and low investment in tolerance can coexist in both ecological and evolutionary time.

### Effects of resources on tolerance

There are two ways to that resources could modify host investment in tolerance. The most direct is if tolerance is itself resource-dependent, for example if increasing resources increases tolerance by making it “cheaper” to invest in tolerance. Existing theory is insufficient to guide predictions here. We show in the Appendix, using the simple model above, that the response of tolerance investment to increased resources is highly sensitive to the shapes of the functions relating resources to tolerance, and tolerance to host fitness (57).

On the other hand, resources can also alter aspects of host physiology or the environment, including by directly changing virulence. These changes will indirectly modify the optimal investment in tolerance. As existing theory typically explores how tolerance changes across gradients of epidemiologically relevant factors, we can use it to understand these indirect effects of resources on tolerance. In particular, we will consider the influence of transmission rate, host lifespan, and host reproduction on tolerance investment. For all of these, theory makes clear predictions and the influence of resources can be inferred straightforwardly.

One of the most commonly explored gradients is transmission rate, β. A universal finding (47, 51, 53, 56, 58) is that, as transmission rate increases, so does investment in either sterility or mortality tolerance. This increased investment in tolerance occurs even as investment in resistance decreases across this gradient (40, 45). These results are entirely intuitive: as transmission rate increases, hosts spend more of their life infected, and thus compensating for the deleterious effects of infection on fitness becomes more important. Resources are likely to affect the transmission rate of many parasites. If parasites are encountered during foraging, either incidentally, as is the case for many parasites in aquatic systems (59), or via intentional ingestion, as is the case for trophically transmitted parasites (60), then transmission rate will be directly related to host foraging rate and thus will be resource-dependent. If increasing resources causes hosts to forage more (or less), theory would predict that investment in tolerance should increase (or decrease). Alternatively, if abundant resources promote host aggregation or reduced host movement, they can also increase transmission via higher contact rates between individuals and/or infected environments (61–63), and hence, increase investment in tolerance.

Increasing host lifespan (either by decreasing the background mortality rate, *m*, or parasite virulence, *v*) is also predicted to increase investment in mortality tolerance (40, 45, 47, 50–53). For sterility tolerance, the results are more complicated, indicting either a unimodal or strictly increasing response of tolerance to host lifespan, depending on the virulence of the parasite (56). Given that increasing resources is likely to reduce the mortality rate from other factors by improving host body condition (64, 65), increasing resources will often increase the investment in tolerance.

The consequences of increasing fecundity on tolerance investment has received only limited theoretical exploration (66). That study varied the birth rate of infected hosts relative to uninfected hosts, *f*, to study how investment in mortality tolerance and other defense strategies varied. They showed that, as the birth rate of infected hosts increased, so did the investment in tolerance, even when increased investment in tolerance compromised investment in resistance mechanisms (42). Again, increased resources is likely to increase investment in tolerance, as infected hosts are more likely to reproduce at near-normal levels when resources are abundant (67). As we show in the Appendix in Supplementary Material, a model incorporating an explicit effect of resources on birth rate would also make the same prediction: if increasing resources increases birth rate, that will also increase investment in tolerance.

The importance of understanding how tolerance will respond to increased resources is magnified by the fact that the evolution of tolerance is often very sensitive to the initial level of tolerance in the population. For example, Miller et al. (53) found that, at an intermediate host lifespan, the host can evolve toward either high tolerance or complete intolerance, depending on the initial level of tolerance in the population. Such bistability between high tolerance and low tolerance strategies is actually a very common finding in studies of tolerance (40, 48, 51, 56), indicating that it is fairly general across a wide range of epidemiological conditions. The implication of such bistability for predicting how resources affect tolerance investment is therefore two-fold. First, if resources are abundant, they may increase the likelihood that tolerance “wins out” over intolerance, as in models showing contingent competition between tolerant and intolerant host populations (51). Second, evolutionary bistability is often characterized by hysteresis, where small changes in the environment can trigger massive changes in the system state. Thus, were a system to start in a bistable region of parameter space where the fitness-maximizing investment in tolerance was very low, an increase in resources could cause the system to pass into a region where the fitness-maximizing investment in tolerance was very high, leading to a sudden jump in investment. Because of the hysteresis, however, a reduction in resources wouldn't necessarily lead to a sudden drop in investment (53).

## Merging empirical and theoretical inferences

As we discuss below, existing theory has three major implications for empirical studies of tolerance. Existing empirical studies have focused on how resources can directly affect host tolerance. Our review of theory suggests that resources may also indirectly affect tolerance by changing the ecological context of host-parasite interactions (e.g., by altering contact rates and, hence, the benefits of investment in tolerance). Human activities are altering the quality, quantity, and distribution of resources available to hosts in the environment (68, 69). This ubiquitous feeding of wildlife by humans, whether intentional or incidental, has a multitude of consequences for wildlife disease (9, 15, 62). The cross-scale effects of anthropogenic resource subsidies are well-described in a recent theme issue of Philosophical Transactions of the Royal Society B (33), but the effects of resources on tolerance (in contrast to effects on resistance) are only discussed in one review (70) and noted as warranting further research in another (9). In particular, a number of studies have documented how anthropogenic resources can promote host aggregation and limit host movement in ways that will increase transmission, and theoretically, investment in tolerance (61–63). Clearly, the study of resource provisioning on other aspects of infection defense (71) are ahead of research on tolerance. Yet, changes in tolerance in response to anthropogenic resource supplementation could have important implications for disease dynamics.

The prediction that mortality tolerance and sterility tolerance can have very different epidemiological and evolutionary trajectories indicates that a critical empirical consideration in studies of tolerance is to carefully diagnose the benefits and costs of tolerance. This is particularly relevant for understanding the influence of resources, as food intake will influence all aspects of an organism's life history, including traits involved in reproduction and survival. Thus, changes in resources may be very likely to influence both mortality and sterility tolerance and, whenever possible, empirical studies should try to quantify both.

In some cases, the measure of tolerance can be cleanly related to either sterility tolerance [e.g., parental provisioning in birds (21–23)] or mortality tolerance [e.g., lifespan of fruit flies (29)], but in many cases, host tolerance is measured via a fitness proxy like body weight that is more challenging to relate to theory (1, 44). There is also the unique issue that there is no universally agreed-upon way to quantify tolerance. A common approach is to quantify some host trait across varying parasite loads, with tolerance quantified as the slope of a regression of trait against load (1, 2), an approach that has attracted criticism (5). However, this means that empirical measures of tolerance have units of things like “body weight per parasite.” Theory, on the other, tends to ignore parasite load, assuming all hosts have equal loads, and measure tolerance as a scalar multiplier on some other trait. Of course, this is a generic problem when trying to relate theory to data, as theoreticians often do not consider how traits are actually measured empirically, and empiricists often do not (or cannot) measure the parameters of a theoretical model. One possible middle ground would be for theory to make more use of models that can account for load, such as classic macroparasite models (72), or nested models (73), and for empiricists to report known relationships between fitness proxies and reproduction and mortality (e.g., if tolerance is measured by body weight, what is the relationship between body weight and reproduction and mortality?).

A further general implication of theory is that tolerance may be difficult to measure (50, 52). For example, if hosts and parasites simultaneously adjust their investment in mortality tolerance (*h*_α_) and virulence (*p*_α_), either coevolutionarily or plastically, infection-induced mortality α may remain constant across environments. This is because increased host investment in mortality tolerance will be countered by increased parasite investment in virulence traits. As hosts increase investment in tolerance, infection-induced mortality decreases; this allows parasites to increase their investment in virulence traits (which typically carry a benefit of increasing transmission, e.g., $\alpha^{\prime }\left(p_{\alpha }\right)>0$ and $\beta^{\prime }\left(p_{\alpha }\right)>0$) without actually increasing infection-induced mortality. Resources may be quite likely to provoke a similar effect; for example, if increasing resources improves investment in mortality tolerance but simultaneously increases parasite abundance within the host (14), the overall change in observed mortality may be negligible. Thus, quantifying or experimentally manipulating parasite abundance will be central to empirically testing the effects of resources on tolerance. Additionally, new tools such as immune gene expression markers of tolerance (74–76) may offer ways to quantify investment in tolerance that are independent of parasite virulence. Finally, a examining tolerance across a range of resource levels ranging from scarce, to normal, to super-abundant will provide much needed insight into the resource-tolerance relationship.

However, it is clear that more theory is needed as well. As our empirical review indicates, increasing resources can either increase or decrease tolerance; our theory review, on the other hand, seems to suggest that the effects of resources on host life history and environment will tend to lead to increasing resources increasing tolerance. The model developed in the Appendix in Supplementary Material is much more nuanced, indicating that this prediction is not nearly so straightforward, especially if resources can directly affect tolerance. However, the model also indicates that predictions will be highly sensitive to the shapes of the functions relating host life history to both tolerance and resources. We hope that the model laid out in the Appendix in Supplementary Material will provide researchers with a jumping-off point for future theoretical work.

As recognition of the importance and frequency of tolerance as a defense strategy grows, a critical next step is to understand variation in tolerance. The studies reviewed here show that resources can affect intra-individual, intraspecific, and interspecific variation in tolerance. They also reveal both the taxa-specific investigations of tolerance (e.g., provisioning behavior in birds, anorexia in flies) and cross-taxa trends that supersede them. For example, in both birds and fruit flies, a low resource diet can improve tolerance (24, 29, 30). Adding resources into existing evolutionary models supports the context-dependent empirical results and provides mechanisms and hypotheses warranting further empirical study. Moreover, these models illustrate the need to quantify tolerance in relation to both mortality and sterility to make accurate ecological and evolutionary predictions. Indeed, now that the effects of resources on tolerance have broadly demonstrated, investigating the ecological and evolutionary consequences of resource-dependent tolerance is a critical next step.

## Author contributions

SB and CC contributed jointly to all parts of the project.

### Conflict of interest statement

The authors declare that the research was conducted in the absence of any commercial or financial relationships that could be construed as a potential conflict of interest.
