# Supplementary material for: Fueling Defense: Effects of Resources on the Ecology and Evolution of Tolerance to Parasite Infection
Source: Front Immunol. 2018 Oct 31;9:2453. doi: 10.3389/fimmu.2018.02453 (PMC6220035; doi:10.3389/fimmu.2018.02453)
Supplement: Supplementary file 1 [file Data_Sheet_1.PDF]

Appendix

We take an adaptive dynamics approach to ask how the evolutionarily stable investment in tolerance changes with resources. Our model is an extension of the simple model introduced in the manuscript (Best et al. 2017). We consider two host populations, a resident (S, I) and mutant (S_m, I_m), that are characterized by traits h and h_m, respectively. Here we assume increasing h represents an increased investment in tolerance that lowers virulence. For an evolutionarily stable tolerance strategy to exist, it is necessary for this investment to carry some cost to the host (i.e., for there to be a trade-off). In general, this could be accomplished by making intrinsic birth rate a, sensitivity to crowding q, or background mortality rate m, a function of h. Here we assume that intrinsic birth rate a is a decreasing function of h.

To study how investment in tolerance changes with resources, we need to consider how resources will enter the model. There are two possibilities. One could explicitly model resource dynamics, allowing resources to be depleted by host ingestion and replenished either by growth or supplementation. This would allow for dynamical feedbacks between host strategy and resources that could potentially drive complex evolutionary dynamics (e.g., Hite and Cressler 2018). More simply, one could treat resources as a parameter, R, of the model. This simpler approach is the one we take here. As noted in the main text, resources can either *directly* affect tolerance investment, or they can *indirectly* affect tolerance investment by affecting other processes in the model.

Resources have a direct effect when tolerance is an explicit function of resources. In this case, we need to define a *tolerance function* $\tau(h, R)$ that is a function of both tolerance investment, h, and resources, R. We assume that $\frac{\partial \tau}{\partial h} > 0$, so that increasing the investment in tolerance will increase tolerance. If increasing resources increase tolerance, the $\frac{\partial \tau}{\partial R} > 0$ as well. The consequence of this resource dependence would be that the same value of τ could be attained at a lower h in a high resource environment.

Resources have an indirect effect on tolerance through their effects on other processes in the model. For example, increasing resources is likely to increase the intrinsic birth rate and decrease both the crowding effect, q, and background mortality rate, m. In our analysis below, we will assume that resources affect intrinsic birth rate, a. Taking into account the preceding discussion of the direct effects of resources on tolerance, we would write intrinsic birth rate as $a(\tau(h, R), R)$. Although unwieldy, this captures the direct effect of resources on tolerance, τ, the effect of tolerance on birth rate ($\frac{\partial a}{\partial \tau} < 0$), the effect of tolerance investment on birth rate ($\frac{\partial a}{\partial h} = \left(\frac{\partial a}{\partial \tau}\right)\left(\frac{\partial \tau}{\partial h}\right) < 0$), and the direct effect of resources on birth rate, separate from resources' effects on tolerance ($\frac{\partial a}{\partial R} > 0$). The full effect of resources on birth rate depends on the sum of these effects: $\frac{da}{dR} = \frac{\partial a}{\partial R} + \left(\frac{\partial a}{\partial \tau}\right)\left(\frac{\partial \tau}{\partial R}\right)$.

Resources may also modify virulence. This effect could be positive, i.e., increasing resources increases virulence, if, for example, virulence depends on pathogen load, and pathogen load is an increasing function of resources (Cressler et al. 2014). It could also be negative, i.e., increasing resources decreases virulence, if, for example, increasing resources reduced the expression of pathogenic phenotypes (e.g., siderophores, Dale et al. 2004). In either case, resources are having a direct effect on tolerance. For example, if increasing resources reduces virulence, then a lower investment in tolerance is required in a high resource environment to achieve the same virulence We write virulence as $\alpha(\tau(h, R), R)$. The effect of tolerance on virulence is given by $\frac{\partial \alpha}{\partial \tau} < 0$; the effect of tolerance investment on virulence is $\frac{\partial \alpha}{\partial h} =$

$\left(\frac{\partial \alpha}{\partial \tau}\right)\left(\frac{\partial \tau}{\partial h}\right) < 0$; and the direct effect of resources on virulence is $\frac{\partial \alpha}{\partial R}$. The full effect of resources on virulence depends on the sum $\frac{d\alpha}{dR} = \frac{\partial \alpha}{\partial R} + \left(\frac{\partial \alpha}{\partial \tau}\right)\left(\frac{\partial \tau}{\partial R}\right)$.

The full model is:

$$\begin{aligned}
\frac{dS}{dt} &= a(\tau(h,R),R)(S+fI) - q(S+fI)(S+I+S_m+I_m) - mS - \beta S(I+I_m) + \gamma I \\
\frac{dI}{dt} &= \beta S(I+I_m) - (\alpha(\tau(h,R),R) + m + \gamma)I \\
\frac{dS_m}{dt} &= a(\tau(h_m,R),R)(S_m+fI_m) - q(S_m+fI_m)(S+I+S_m+I_m) - mS_m - \beta S(I+I_m) + \gamma I_m \\
\frac{dI}{dt} &= \beta S(I+I_m) - (\alpha(\tau(h_m,R),R) + m + \gamma)I
\end{aligned} \tag{1}$$

The analysis of this full model is unwieldy, to say the least, but we will illustrate how such an analysis would proceed by considering a slightly simplified model. In particular, we will assume that resources have no direct effects on tolerance, but can affect both intrinsic birth rate and virulence. In this case, we can write intrinsic birth rate as $a(h,R)$, with $\frac{\partial a}{\partial h} < 0$ and $\frac{\partial a}{\partial R} > 0$. We can write virulence as $\alpha(h,R)$, with $\frac{\partial \alpha}{\partial h} < 0$ and $\frac{\partial \alpha}{\partial R}$ left unspecified for generality.

Following standard practice for an adaptive dynamics analysis, we assume that the resident host is at its ecological equilibrium and ask whether the mutant host can invade from rarity. Mathematically, whether the mutant can invade is determined by the eigenvalues of the Jacobian matrix of partial derivatives, evaluated at the equilibrium $(S^*, I^*, 0, 0)$. That 4x4 Jacobian will have a block triangular structure: the upper-left 2x2 submatrix determines the stability of the resident-only system (so its eigenvalues will be negative) and the lower-left submatrix is 0, so the eigenvalues of the lower-right submatrix will determine whether the mutant can invade. This matrix is:

$$J = \begin{pmatrix} a(h_m,R) - q(S^*+I^*) - m - \beta Q^* & f\big(a(h_m,R) - q(S^*+I^*)\big) + \gamma \\ \beta Q^* & -(\alpha(h_m,R) + m + \gamma) \end{pmatrix} \tag{2}$$

Applying the Next Generation Matrix theorem (Hurford et al. 2010), the mutant will be able to invade if

$$\begin{aligned} r_m = (a(h_m,R) - q(S^*+Q^*) - m - \beta Q^*)(\alpha(h_m,R) + m + \gamma) \\ + \beta Q^*\big(f\big(a(h_m,R) - q(S^*+I^*)\big) + \gamma\big) > 0. \end{aligned} \tag{3}$$

Possible endpoints of evolution occur at tolerance investment strategies $(h_m = h = h^*)$ that cause the fitness gradient $\left.\frac{\partial r_m}{\partial h_m}\right|_{h_m=h=h^*}$ to vanish (keeping in mind that all derivatives are evaluated at h^*),

$$\left.\frac{\partial r_m}{\partial h_m}\right|_{h_m=h=h^*} = (\alpha(h^*,R) + m + \gamma + f\beta Q^*)\frac{\partial a}{\partial h} + (a(h^*,R) - q(S^*+Q^*) - m - \beta Q^*)\frac{\partial \alpha}{\partial h} = 0. \tag{4}$$

Since both $\frac{\partial a}{\partial h} < 0$ and $\frac{\partial \alpha}{\partial h} < 0$, for an ES to exist it must be the case that $(a(h^*,R) - q(S^* + Q^*) - m - \beta Q^*) < 0$.

h^* will be evolutionarily stable (i.e., a fitness maximum) if $\left.\frac{\partial^2 r_m}{\partial h_m^2}\right|_{h_m=h^*} < 0$, where

$$\left.\frac{\partial^2 r_m}{\partial h_m^2}\right|_{h_m=h=h^*} = 2\frac{\partial a}{\partial h}\frac{\partial \alpha}{\partial h} + (\alpha(h^*,R) + m + \gamma + f\beta Q^*)\frac{\partial^2 a}{\partial h^2} + (\alpha(h^*,R) - q(S^* + Q^*) - m - \beta Q^*)\frac{\partial^2 \alpha}{\partial h^2} \tag{5}$$

Since increased investment in tolerance reduces both virulence and intrinsic birth rate $\left(\frac{\partial a}{\partial h} < 0, \frac{\partial \alpha}{\partial h} < 0\right)$ and $(\alpha(h^*,R) - q(S^* + Q^*) - m - \beta Q^*) < 0$, the second partial derivatives could be almost any sign, implying that the shape of the functional relationship between birth rate, virulence, and tolerance is fairly unconstrained. Keeping in mind that $\frac{\partial^2 a}{\partial h^2} = 0$ implies that birth rate decreases linearly with tolerance, $\frac{\partial^2 a}{\partial h^2} < 0$ implies that birth rate decreases at an accelerating rate with tolerance, and $\frac{\partial^2 a}{\partial h^2} > 0$ implies that birth decreases at a decelerating rate with tolerance, there are a few constraints and conclusions that can be drawn.

1. Either virulence or birth rate must be a nonlinear function of tolerance, otherwise evolutionary stability is impossible.
2. If virulence is a linear function of tolerance, then birth rate must decrease at an accelerating rate with tolerance.
3. If birth rate is a linear function of tolerance, then virulence must decrease at a decelerating rate with tolerance.

Evolutionary stability is most likely if birth rate decreases at an accelerating rate and virulence decreases at a decelerating rate: in other words, if the costs of tolerance increase faster than the benefits. Assume that we are only interested in evolutionarily stable (ES) tolerance investments ($h_m = h = h^*$). It is clear that the value of any ES will implicitly depend on resources, R, in that altering R will alter h^*. We can write h^* as a function of R and implicitly differentiate the ES condition with respect to R to try to gain insight into how changing R will affect ES tolerance. That gives us an expression containing the derivative $h^{*\prime}(R)$. Solving for $h^{*\prime}(R)$, we arrive at an expression whose sign tells how the ES investment in tolerance changes with resources (keeping in mind that all derivatives are evaluated at $h = h^*$):

$$h^{*\prime}(R) = \frac{-\frac{\partial a}{\partial h}\frac{\partial \alpha}{\partial h} - \frac{\partial a}{\partial R}\frac{\partial \alpha}{\partial R} - (\alpha(h^*,R) + m + \gamma + f\beta Q^*)\frac{\partial^2 a}{\partial h \partial R} - (\alpha(h^*,R) - q(S^* + Q^*) - m - \beta Q^*)\frac{\partial^2 \alpha}{\partial h \partial R}}{2\frac{\partial a}{\partial h}\frac{\partial \alpha}{\partial h} + (\alpha(h^*,R) + m + \gamma + f\beta Q^*)\frac{\partial^2 a}{\partial h^2} + (\alpha(h^*,R) - q(S^* + Q^*) - m - \beta Q^*)\frac{\partial^2 \alpha}{\partial h^2}}.$$

Notice that the denominator of this expression is the evolutionary stability condition, meaning that we know that it will be negative. $\frac{\partial a}{\partial h}\frac{\partial \alpha}{\partial h}$ will be positive, as increasing investment in tolerance decreases both intrinsic birth rate and virulence; $\frac{\partial a}{\partial R}\frac{\partial \alpha}{\partial R}$ could be negative (if increasing resources increases virulence), positive (if increasing resources decreases virulence), or zero (if resources have no direct effect on virulence), as increasing resources will increase birth rate. The mixed partial derivatives have indeterminate signs. This suggests that, in general, it is possible to choose functional forms such that tolerance investment can increase or decrease with resources.

However, we can consider one case more carefully: assume that resources have no effect on virulence. Then $\frac{\partial\alpha}{\partial R} = \frac{\partial^2\alpha}{\partial h\partial R} = 0$; if $\left(-\frac{\partial a}{\partial h}\frac{\partial\alpha}{\partial h} - (\alpha(h^*, R) + m + \gamma + f\beta Q^*)\frac{\partial^2 a}{\partial h\partial R}\right) < 0$, then increasing resources will increase tolerance (because the denominator above is negative). This is guaranteed if $\frac{\partial^2 a}{\partial h\partial R} > 0$.

As an example of the potential utility of this modeling framework, we can use it to compare with a prediction made in the main text. In particular, using the function giving the effect of tolerance investment on intrinsic birth rate in Best et al. (2017), and assuming a linear effect of resources on birth (not included in the original paper), we find that $\frac{\partial^2 a}{\partial h\partial R} > 0$. As expected, resources increasing birth rate would also lead to an increase in tolerance, as predicted by Best et al. (2017).

References:

Best A, White A, & Boots M. (2017). The evolution of host defence when parasites impact reproduction. *Evolutionary Ecology Research, 18,* 393–409.

Cressler, C. E., W. A. Nelson, T. Day, & McCauley, E. (2014). Disentangling the interaction among host resources, the immune system, and pathogens. *Ecology Letters, 17,* 284-293.

Dale, S. E., Doherty-Kirby, A., Lajoie, G., & Heinrichs, D. E. (2004). Role of siderophore biosynthesis in virulence of *Staphylococcus aureus*: identification and characterization of genes involved in production of a siderophore. *Infection and Immunity, 72,* 29-37.

Hite, J. L. & Cressler, C. E. (2018). Resource-driven changes to host population stability alter the evolution of virulence and transmission. *Philosophical Transactions of the Royal Society B.*, *373,* 20170087.

Hurford, A., Crownden, D., & Day, T. (2010). Next-generation tools for evolutionary invasion analyses. *Journal of the Royal Society Interface*, 7, 561-571.
